# Economic cost of not meeting the 24-h movement guidelines in china: Research gaps and recommendations

**DOI:** 10.1016/j.smhs.2024.05.003

**Published:** 2024-05-21

**Authors:** Sitong Chen, Jie Feng, Yang Liu

**Affiliations:** Institute for Health and Sport, Victoria University, VIC, Australia; Department of Sports Science and Physical Education, The Chinese University of Hong Kong, Hong Kong SAR, China; School of Physical Education, Shanghai University of Sport, Shanghai, China; Shanghai Research Centre for Physical Fitness and Health of Children and Adolescents, Shanghai University of Sport, Shanghai, China

**Keywords:** Movement behavior, Cost analysis, Policy, Practice

## Abstract

It is well-known that not meeting the **24-hours(h)** movement guidelines, including insufficient physical activity (PA), excessive sedentary behavior (SB), inadequate sleep duration, and their combinations, are independent risk factors for noncommunicable diseases (NCDs). The prevalence of not meeting the guidelines is high across the world, especially in China where has one of the largest population. Some studies have estimated the economic cost of insufficient PA in China, which is useful to guide policymakers to develop and implement effective health actions. However, several research gaps should be discussed and addressed for better evidence base and decision making. This commentary aims to provide a research insight into gaps and recommendations related to the analysis of economic cost of not meeting the 24-h movement guidelines. Some major research gaps can be indicated, including less research attention on excessive SB and inadequate sleep, limited evidence regarding NCDs associated with not meeting all 24-h movement guidelines considered in economic cost analysis, absence of evidence on estimated cost of not meeting the guidelines, and the adherence to methodological guide. Future research is required to address the gaps to guide effective health policy development in China. We hope that this commentary can play an important role in updating research evidence and advancing policy practice.

## Abbreviation list

NCDs: Noncommunicable diseases
PA: Physical activity
SB: Sedentary behavior
VIRTUE: Viable Integrative Research in Time-Use Epidemiology

## 1 Noncommunicable diseases and not meeting the 24-hour movement guidelines

Noncommunicable diseases (NCDs) is responsible for 86% of all deaths in the world according to the World Health Organization data.^1^ This number suggests the substantial health burdens from NCDs on global and national healthcare systems. Owing to the adverse effects,^2^ developing effective NCDs prevention and control plans is considered important to create “a healthy society for all”. A growing body of evidence demonstrates the strong associations between unhealthy 24-hour (h) movement behaviors (i.e., in insolation or combination) and increased risks for NCDs.^3^, ^4^, ^5^, ^6^ Movement behaviors consist of physical activity (PA), sedentary behavior (SB) and sleep,^7^ making up a 24-h day. Integrating all movement behaviors throughout the day recognizes “the whole day matters” in health promotion given their interdependencies. For this, some national organizations have developed the 24-h movement guidelines^8^ from the perspective of integrative approaches. Thus, insufficient PA, excessive SB and inadequate sleep, as well as their combination(s), can be operationalized as not meeting the 24-h movement guidelines.^9^^,^^10^

China experiences the surge of NCDs^11^, ^12^, ^13^, ^14^, ^15^ and high prevalence of insufficient PA, excessive SB and inadequate sleep duration.^16^, ^17^, ^18^ Strong evidence has been well-established on the associations of insufficient PA, excessive SB, and inadequate sleep duration with a range of NCDs in Chinese population.^13^^,^^14^ For example, insufficient PA has been identified as a major risk factor for type 2 diabetes in Chinese adults.^11^^,^^19^ If insufficient PA would be eliminated, 0.61 years of gain in life could be made for Chinese population.^20^ Owing to increasing NCDs and the prevalence of not meeting the 24-h movement guidelines in Chinese population,^11^^,^^13^ Chinese government has issued a series of NCDs prevention and control programs, such as the *Healthy China 2030 Blueprint*.^21^ Given the health consequences, it is not surprising that lowering the prevalence of not meeting the 24-h movement guidelines contributes to save economic burden of NCDs.

Some previous studies have estimated the economic cost of unhealthy movement behaviors in China,^22^, ^23^, ^24^, ^25^ which provides evidence base to inform health policy development and implementation. However, there are some gaps on the economic cost analysis of not meeting the 24-h movement guidelines in China. In the following sections, we will first present some evidence showing the substantial economic cost of not meeting the 24-h movement guidelines (i.e., in insolation) and then highlight the resultant research gaps for future updates.

## 2 Current evidence in the literature

A large number of studies have estimated the economic cost of insufficient PA,^26^, ^27^, ^28^, ^29^, ^30^, ^31^, ^32^, ^33^ excessive SB,^33^, ^34^, ^35^, ^36^, ^37^ and inadequate sleep duration^38^^,^^39^ in insolation, even though these studies were primarily based on high-income or western countries.^40^ All the studies indicated that non-adherence to either the individual guideline caused substantial economic losses, accounting for a large proportion of national healthcare cost.^41^ Given that such economic cost analyses can clearly demonstrate economic savings of taking actions and inform public health importance of healthy lifestyles, it needs to be continuously conducted.^41^^,^^42^

Compared to the abundance of evidence from western countries, relatively less attention is placed on China's situation. In the literature, a few studies reported the economic losses.^22^, ^23^, ^24^, ^25^ Popkin et al.,^22^ using the data in 2000, assessed the economic cost of insufficient PA and the result was 1.6 billion US $. Zhang et al.^23^ estimated that insufficient PA was responsible for economic cost of 6.7 billion US $ in 2007. Ding et al.^24^ conducted the first international study to estimate the economic cost of insufficient PA, and they found the cost was 4.9 billion International$ in 2013. The three studies used the population attributable fraction approach for estimation at the macrolevel.^22^, ^23^, ^24^ Interestingly, according to the RAND Europe Company, 100–146 billion US $ can be saved if insufficient PA would be eliminated and the prevalence of sufficient PA can be increased by 20%.^43^ One recent study demonstrated that incidence of insufficient PA from 2020 to 2030 would result in a loss of 322 billion International $.^44^ Except for the macro-level estimation, there was one study using individual-level data on economic cost of insufficient PA.^25^ The authors suggested that less PA was associated with increased out-of-pocked money of health expenditure in Chinese older adults.^25^ In sum, regardless of study year and estimation approach, these studies all drew that insufficient PA considerably contributed to substantial economic costs in China.

Although the previous studies have highlighted enormous economic losses in China, some noticeable research gaps still remain. These research gaps must be addressed to strengthen the research evidence base and inform policy practice in China.

## 3 Research gaps and future recommendations

### 3.1 Less research attention on excessive SB and inadequate sleep

In the 24-hour movement behavior research field, (in) sufficient PA is still the most-studied research topic. Such a scenario is observed in the research on economic cost. To the best knowledge, four studies have reported the economic cost of insufficient PA in China since 2006.^22^, ^23^, ^24^, ^25^ By contrast, only one study examined economic cost of excessive SB,^25^ and no studies assess inadequate sleep in China. This lack of information hampers the public and the relevant policymakers to understand the economic and societal impacts of excessive SB and inadequate sleep duration. Ample evidence has confirmed the associations of excessive SB^19^ and inadequate sleep duration with several NCDs.^45^, ^46^, ^47^, ^48^ According to the Framework for Viable Integrative Research in Time-Use Epidemiology (VIRTUE),^49^ one important research area is to assess the outcomes of time-use components, including PA, SB and sleep. Guided by this research framework, theoretically, it is essential to assess the economic cost of excessive SB and inadequate sleep duration individually. Addressing this gap is crucial for researchers and policymakers to develop interventions aimed at reducing excessive SB and inadequate sleep duration, and to target key elements to improve public health.

### 3.2 Limited NCDs included in economic cost analysis

In the published literature, the research on economic cost considered a limited number of NCDs associated with insufficient PA. For example, Zhang et al.^23^ included five major NCDs in their study. Similarly, Ding et al.^24^ analysed six NCD indicators to estimate the economic cost. According to the current evidence from systematic review, it has been demonstrated that not meeting the 24-hmovement guidelines in insolation or in combination is associated with a variety of NCDs than those studies included,^23^^,^^24^ such as mental health outcome (e.g., depression); therefore, the actual cost when including all confirmed NCDs would be much higher.^42^ Addressing this gap, policymakers could develop more effective strategies to promote healthy 24-h movement behaviors, aimed to prevent NCDs and improve health and well-being in the population. For these reasons, future studies should consider NCDs associated with not meeting the 24-h movement guidelines as many as possible for more accurate estimations. To achieve this, at least two pieces of information, including risk of all NCDs associated with not meeting the guidelines, and expenditure of all NCDs, are necessarily required. In consideration of the information requested for analysis, more details, such as the relative risk derived from longitudinal studies, a clear definition of types of expenditure (e.g., medical cost, healthcare cost, gain in gross domestic production), should be operationalized prior to analysis.

### 3.3 Estimated cost of not meeting the 24-h movement guidelines

Considering the co-dependence of PA, SB and sleep in the nature of time-use epidemiology,^49^ economic cost of combinations of insufficient PA, excessive SB and inadequate sleep should be prioritised. Estimating cost associated with not meeting the 24-h movement guidelines is crucial for policymakers.^42^ By understanding cost burden of unhealthy behaviors such as insufficient PA and/or sedentary lifestyle, policymakers can better adjust allocation of healthcare resources and services. Conducting such an analysis is helpful for policymakers to understand the importance of comprehensive actions against not meeting the 24-h movement guidelines. However, to our knowledge, an important research gap is that no research examines the economic cost of thenon-adherence to the combinations (e.g., both insufficient PA and excessive SB). Also, when contemplating the VIRTUE framework, it theoretically provides foundation to assess the economic cost of not meeting the 24-h movement guidelines, but this kind of study would need to exclude “overlapping effect” in estimation. In addition, if considering the nature of co-dependence for time spent in PA, SB and sleep,^49^^,^^50^ how their reallocations affect economic cost or contribute to economic savings is another research gap but such analyses need individual-level data.

### 3.4 Adherence to the guide to improve economic analysis

It is important for researchers to adopt robust, standardized, and transparent methods when estimating the economic cost of NCDs and the risk factors.^40^ However, across the studies based on China's data,^22^, ^23^, ^24^ methodological inconsistencies were observed. Divergent methods may reduce credibility in decision making; therefore, a standardized guideline would be needed to be developed and used for research. There are no currently standardized methodological guidelines for economic cost analysis as it is challenging to fully standardize the related methods given the different study objectives.^40^ The Checklist for reporting estimates of the economic costs/burden of risk factors was developed in 2017 by Ding et al.^40^ The checklist can be used as a guide for improving methodological rigour and reporting quality to reach specific research needs and help conduct decision making. Based on this, when assessing the economic cost in China, researchers are suggested to adhere to the checklist. In addition to this, more considerations on the conservative estimation must be mentioned. In the literature, most studies found the estimated economic cost conservative values,^40^ which may be lower than the actual levels. The main reasons for the underestimation include: (1) underestimated prevalence of insufficient PA, excessive SB and inadequate sleep duration, as well as their combinations because of the use of self-reported measures; (2) limited data sources of different kinds of costs associated with NCDs. These two reasons are mainly responsible for the conservative estimation. By addressing these, the credibility of estimation of economic cost can be more effective in policy development and implementation. Therefore, adopting standardized methods for estimating the economic cost of not meeting the 24-h movement behaviors in China is crucial for enhancing methodological consistency and transparency, facilitating comparisons and synthesis of findings, improving research reproducibility, and enhancing policy effectiveness.

## 4 Summary

Economic analysis plays a crucial role in guiding public health policies in China, especially as the promotion of healthy lifestyles and the prevention of NCDs become increasingly important. The growing significance of research effort into the economic impacts of 24-h movement behaviours highlights the need for robust analytic capabilities to inform policy decisions. By addressing existing research gaps, we can enhance the effectiveness of public health decision-making in China, fostering the implementation of evidence-based interventions, and enhance policies effectiveness. This commentary aims to advocate for strengthened research efforts into health economic analysis in China, underscoring the potential benefits of endeavors for public policy and health promotion.

## Conflict of interest

The authors declare no competing interest.

## CRediT authorship contribution statement

**Sitong Chen:** Writing – review & editing, Writing – original draft, Validation, Supervision, Project administration, Methodology, Conceptualization. **Jie Feng:** Writing – review & editing, Writing – original draft. **Yang Liu:** Writing – review & editing, Validation.
